# Rectifying COVID-19 disparities with treatment and vaccination

**DOI:** 10.1172/jci.insight.147800

**Published:** 2021-02-22

**Authors:** John M. Carethers

**Affiliations:** Department of Internal Medicine, Department of Human Genetics, and Rogel Cancer Center, University of Michigan, Ann Arbor, Michigan, USA.

## Introduction

Severe acute respiratory syndrome coronavirus 2 (SARS-CoV-2) is a novel virus that causes a spectrum of disease in humans called coronavirus disease 2019 (COVID-19). Those infected display a range of disease from completely asymptomatic to mild, moderate, or severe manifestations that can lead to death (1, 2). Moreover, infected persons spread virus even prior to displaying overt symptoms (3). SARS-CoV-2 gains entry to host cells via its spike protein, which binds host angiotensin-converting enzyme type 2 (ACE2) receptors that are heavily expressed in nasal airway and lung tissue in combination with host neuropilin-1 receptors. Subsequent cleavage of the spike protein by the peptidase furin is then followed by virus-host membrane insertion by the human protease TMPRSS2 (4–6). Although the pulmonary tract is the principal organ system affected by this virus and the site of viral pneumonia, other organ systems may also be sites of primary SARS-CoV-2 infection and others can be secondarily compromised with severe COVID-19 disease (2, 7, 8).

Because COVID-19 is a novel human disease, people have no protective immunity, and the disease has become an ongoing pandemic for the worldwide population beginning in 2020 and into 2021. The US CDC recommends use of masks, social distancing, and hand hygiene as major methods to prevent spread of infection throughout the population. Certain groups within the population are at higher risk for infection and/or severe disease, including those in older age groups and those who are male (9). Health comorbidities have also emerged as a key factor for higher risk for COVID-19 (9). The CDC lists the presence of cancer, chronic kidney disease, chronic obstructive pulmonary disease (COPD), heart conditions, immunocompromised state, obesity, pregnancy, sickle cell disease, smoking, and type 2 diabetes as increased risk factors for severe COVID-19 (10). Additionally, the presence of several other conditions, including asthma, cerebrovascular disease, cystic fibrosis, hypertension, dementia, liver disease, pulmonary fibrosis, thalassemia, and type 1 diabetes, may place individuals at increased risk for COVID-19 (10). One rationale for the increased COVID-19 risk in men is higher expression of androgen-driven ACE2 and/or TMPRSS2 expression (9). Several comorbidities are also associated with increased ACE2 and TMPRSS2 expression, although the mechanism by which these conditions induce expression of these receptors is not clear (9, 11, 12). Moreover, the presence of comorbidities increases with age and may be the principal driving force for higher COVID-19 risk in older populations (9, 13).

Sadly, the distribution of COVID-19 infection, hospitalization, and death varies by race and ethnicity in the US (and UK), creating a disparity for this disease (14, 15). For American Indian/Alaskan Native populations, cases, hospitalization, and death occur at rates that are 1.8, 4.0, and 2.6 times, respectively, of those that occur in White populations. African American populations exhibit cases, hospitalization, and death rates that are 1.4, 3.7, and 2.8 times the rates of White populations, whereas cases, hospitalizations, and death rates in Latin American populations are 1.7, 4.1, and 2.8 times the rates of White populations (14). For Asian populations, the rates for hospitalizations and death are similar to those of White populations (14). Early surges during the COVID-19 pandemic demonstrated a relative risk of death for African American populations versus White populations of 3.57 (95% CI, 2.84–4.48) and a relative risk of death for Latin American populations of 1.88 (95% CI, 1.61–2.19) (16). Unlike the higher levels of ACE2 and/or TMPRSS2 expression in men and those with health comorbidities, the driver for racial and ethnic disparities with COVID-19 appears principally to be socioeconomic inequalities that are exacerbated by the existence of comorbidities (9, 17). Death from COVID-19 varies by US county, with fewer deaths in counties populated by those who are college educated, hold medical insurance, and have higher incomes, as compared with counties with more diverse populations that are associated with poor grocery mobility (food deserts), poor work mobility (e.g., need for public transportation), and higher SARS-CoV-2 infection rates (17). Health comorbidities are more prevalent in specific diverse populations, thus increasing the risk for severe COVID-19 (18). By the age of 65 years, 40% of the African American population demonstrates 3 or more comorbid risk factors, compared with 29% of White and 28% of Latin American populations (18). Even between the ages 45 and 64 years, 23% of the African American population shows 3 or more comorbid risk factors, compared with 13% of White and 11% of Latin American populations (18). The increased frequency of comorbidities is a consequence of socioeconomic inequality. Over a lifetime, persistent lower socioeconomic inequality determines where one resides (lower-income and high-density neighborhoods with food deserts and limited preventive medical care); the type of job held (lower paying jobs); the access and affordability of fresh food versus eating a high-fat, high-caloric, low-fiber diet; the use of alcohol and tobacco; and lower physical activity (lack of parks or open spaces). These conditions create biophysiological consequences, including alteration of the gut and pulmonary microbiomes, higher systemic inflammation, and relative compromised immunity. These biophysiological alterations in turn create the conditions for development of comorbidities, such as cancer, obesity, diabetes, hypertension, COPD, and cardiovascular and kidney disease (9), These comorbidities are the exact high-risk conditions for COVID-19. The risk for COVID-19 in racial and ethnic groups parallels with risk for comorbidities, particularly cancer (19–21), all of which seem to have many of their roots in socioeconomic inequities.

The devastating effect of COVID-19 in diverse populations has worsened the already lower baseline economic vitality of these populations. For instance, African American populations are disproportionately represented among essential workers in janitorial, food, and transportation industries, which were initially exempted from shelter-in-place mandates during pandemic surges (22). African American and Latin American households tend to have a larger number of people relative to house size, which undermines social distancing efforts, and are more likely to utilize public transportation (22, 23). With COVID-19, these same populations were also more likely to take a pay cut or lose their job because of the pandemic, and both Latin American and African American populations are much less likely to have “rainy day” funds (24). Overall, the socioeconomic inequalities, coupled with increased health comorbidities, put these populations at higher risk for infection and severe COVID-19; and with the pandemic, the socioeconomic inequalities are widening, fueling an increased vulnerability cycle for infection and severe disease with persistent and worsening socioeconomic conditions (9).

As mentioned above, prevention techniques to avoid exposure to COVID-19 are the recommended best approach to stem infection (Table 1). For some diverse populations, despite utilizing preventive measures in many cases, the circumstances of living conditions and employment make complete avoidance challenging. Vaccination to gain adaptive immunity to the SARS-CoV-2 spike protein (which all current vaccines are based on) is an excellent tool for prevention once it is available to the entire population and a majority becomes inoculated. Once infected (without immunity), a person may be asymptomatic, and without isolation precautions, could continue to spread the virus to others. The risk is higher in specific diverse populations that present with disease, ranging from mild to moderate to severe (Table 1). Equality in treatment of racial and ethnic minorities infected with COVID-19, as well as equal access to vaccination, should mitigate disparities for infection and COVID-19 outcomes.

## Hospitalization and treatment

Treatment recommendations for patients with mild, moderate, and severe COVID-19 are listed in Table 1 (25). In particular, data for patients with severe COVID-19 demonstrate the benefit of supplemental oxygenation, the steroid dexamethasone, and the antiviral remdesivir (25). Most hospitals have access to dexamethasone and oxygen to provide to patients; however, remdesivir is not as abundant and is costly (>$3100 per patient). Therefore, despite its beneficial effects for patients with severe COVID-19, remdesivir is not readily available and leaves questions as to what populations have access. For example, do critical access hospitals and hospitals that serve indigent patients have adequate supplies of the drug, as compared with large hospitals? With the need for hospitalization of those with severe COVID-19, particularly in areas where infection rates are very high and hospitals become full due to smaller numbers of licensed beds, can patients from diverse or indigent communities be transferred to other hospitals that are not full for care if needed? With moderate COVID-19, some patients with comorbidities have access to SARS-CoV-2 antibody or convalescent plasma therapy that may keep them from progressing to severe disease requiring oxygenation and hospitalization. Do more indigent communities have access to this expensive therapy? To date, these are questions that cannot be answered completely; assessing these questions with data would be the first step towards any demonstration for equity of care in diverse communities.

Data demonstrate that higher proportions of African American patients have been principally hospitalized in large US metropolitan areas, including New York City, Detroit, Chicago, Philadelphia, Atlanta, New Orleans, Newark, and Washington, DC (26). Among COVID-19–positive patients, African American populations showed twice the rate for hospitalization compared with White populations (27), similar to that observed for Black patients in the UK compared with White patients (OR 2.4; 95% CI, 1.5–3.7) (15), even after adjustments for age, sex, insurance, and comorbidities. Studies show that residents of low-income areas, from low socioeconomic class, and/or with comorbidities were at substantially higher risk for COVID-19 hospitalization (15, 27, 28). However, once hospitalized, there is no evidence for a disparity for death due to COVID-19, with HRs for death similar between 3481 COVID-19–positive African American and White patients (HR, 1.14; 95% CI, 0.84–1.38) (27) and among a cohort of 11,210 COVID-19–positive African American and White patients (HR 0.93; 95% CI, 0.80–1.09) (28). These data suggest that while more African American people may contract COVID-19, once hospitalized, the risk of death is no different than that of White American populations in similar hospital settings.

There are limited data published examining COVID-19 outcome in different hospital settings, such as critical access hospitals, or comparing large hospitals with smaller community hospitals. It is not known if access to expensive medications before or during hospitalization varies in these settings, and/or if the approach to care is identical, and/or if limited licensed available beds pose problems for in-hospital access and care in specific communities. Care by those from similar backgrounds helps exchange cultural customs and values and helps patients adhere to treatment recommendations and improve patient experience (29). However, there is a dearth of future physicians in the pipeline from populations underrepresented in medicine that will continue to curtail diverse representation at academic, large, and small hospitals (30–33). Additionally, a recent study suggests that African American patients had three times the frequency of occult hypoxemia via pulse oximetry compared with White patients (34). In this COVID-19 era, where there is reliance on pulse oximetry to determine if one has severe disease requiring hospitalization, some African American patients with COVID-19 could be sent home with occult hypoxemia, thus unknowingly increasing the risk for worsening of disease, nontreatment, or for an out-of-hospital COVID-19 death. Furthermore, many diverse communities have limited access to routine, preventive care, which is more adversely disrupted in these communities with COVID-19 (19, 35).

## Vaccination

The ultimate prevention of an infectious disease is through vaccination (Table 1); never in the history of humans has a vaccine been developed as rapidly as the COVID-19 vaccine, making it the first-ever vaccine that may directly affect the pandemic stage of an infectious disease. The scale and timeframe for vaccination in the US and the world is unprecedented in nature and will require multiple strategies to immunize all populations, much like the approaches for cancer screening across diverse populations (35, 36). Vaccination for influenza has been around for years and is an annual process, yet influenza vaccination rates during 2018–2019 were lower in African American (39.4%), Latin American (37.1%), and American Indian/Alaskan Native populations (37.6%) as compared with Asian (44.0%) and White populations (48.7%) (37). The US and the world have an obligation to ensure that the reach of COVID-19 vaccination is far and wide and gets to all populations fairly.

The pandemic that ensued from the novel SARS-CoV-2 virus has pushed creativity and empowered governments and companies to accelerate production of an effective vaccine, with companies who either performed or announced phase III trials listed in Table 2. The new technology of an mRNA vaccine made its debut with COVID-19, while other more traditional technologies for a vaccine are all in development (Table 2). Interim analyses of some late-phase trials of vaccines reveal prevention efficacies from COVID-19 infection and illness as high as 95%, with the new mRNA vaccines showing the highest effectiveness to date (Table 2). As of December 30, 2020, two vaccines have been approved in the US (BNT162b2 and mRNA-1273) and two have been approved in the UK (BNT162b2 and AZD1222), while other vaccines have been approved in Russia, China, the United Arab Emirates, and other countries, generally through emergency use authorization because of the pandemic and not through full government approval (38–45). BNT162b2, mRNA-1273, and AZD1222 have generally proven effective immunogenicity and are safe from causing any severe symptoms in early- and late-phase trials (46–48). Most of the vaccines require two doses for the best efficacy, while some are designed to be administered as one injection (Table 2). Only certain vaccine trials — through interim analyses of phase III trials — have reported the racial/ethnic makeup of trial participants (Table 2). Many of the vaccines are currently being evaluated in phase III trials in South America, the Middle East, Asia, and Africa, but racial and ethnic breakdown for participants in those trials has not yet been reported. The inclusion rates for African American and American Indian/Alaskan Native populations in trials to date in the US lag behind the makeup of the general population, despite these communities being the hardest hit with severe COVID-19 and death (Table 2).

Initially, the number of vaccine doses will be limited due to constraints in production. The Advisory Committee on Immunization Practices (ACIP) that advises the CDC recommends a phased approach to prioritize people for vaccination: phase Ia includes health care personnel and residents of long-term care facilities; phase Ib includes persons aged ≥75 years and frontline essential (nonhealth) workers; phase Ic includes persons aged 65–74 years and persons aged 16–64 years with high-risk medical conditions (49) as well as essential workers not recommended for vaccination in phase Ib; and phase II includes all other persons aged ≥16 years not already recommended for vaccination in phases Ia, Ib, or Ic (50). In theory, the inclusion of frontline essential workers may capture some racial and ethnic populations for vaccination during the early phases of vaccine rollout, as many are employed in essential industries; however, it is not clear if these populations have the same prioritization if they are unemployed.

There will be several challenges to vaccinating diverse populations for COVID-19. Some vaccines require extreme cold for transport and storage, a challenge for some locations, including safety net hospitals or outpatient centers that might lack cold-storage freezer units. There remains some fear of vaccination in the general population that is more heightened in diverse populations, particularly in the African American community, due to past transgressions of trust in clinical trials. As mentioned above, the representation of diverse populations in many medical clinical trials, including those for the COVID-19 vaccine, often does not mirror the diversity seen in the general population. Visible diversity of vaccination participants will be highly important to convince others to take the vaccine. Some people are not convinced of the stated safety of the vaccines, and this doubt remains high within diverse communities. Several vaccines require a second injection weeks after the first injection; while there may be some immunity gained after one dose, optimum immunity requires two doses. Therefore, it will be challenging but paramount to ensure that underinsured and/or diverse communities receive at least one dose — let alone come back for a second dose— to halt the pandemic in these communities.

## Mitigation strategies

As the superb effectiveness of navigation for colorectal cancer screening negates observed disparities in cancer incidence and mortality (51), similar strategies can be applied to COVID-19 infection and prevention. Indeed, such “navigation” of each racial or ethnic community with up-to-date information, while correcting misinformation, gains trust over time. Empowered and visible community leaders need to be servant leaders and set an example. Legislation that provides financial support to local communities, including critical access hospitals and community centers as well as individuals who have become unemployed, may help control COVID-19 infection as well as provide continued medical services in the community. There will be a need to be equitable in the distribution of vaccines, and one important goal will be to aim for each community to reach levels of potential herd immunity at the same time as the rest of the US population. Several mitigation strategies are listed in Table 3.

## Conclusions

There is observed disparity in COVID-19 infections and death, with African American, Latin American, and Native American/Alaskan Native populations showing excess rates of infection and death compared with White populations in the US. The disparity is largely driven by longstanding socioeconomic inequalities and the simultaneous, consequential acquirement of health comorbidities that greatly increase the risk for severe COVID-19 infection, similar to data observed for Black populations in the UK. There are only limited data regarding whether or not difference in treatment or hospital location contributes toward the observed disparity, but in large hospital settings, once hospitalized, death rates from COVID-19 appear the same between African American and White populations. Prevention through immunization with recent government-authorized COVID-19 vaccines will be critical for protection from infection. Convincing communities of color to fully participate in the mass COVID-19 vaccination program will take effort from national and local leaders to adequately and visibly demonstrate the safety and needed protection from acquiring the virus. This could and must be done in an equitable manner for these communities hard hit by COVID-19, and each community should be targeted to reach herd immunity at the same rates as other communities with vaccine availability.

## Figures and Tables

**Table 1:**
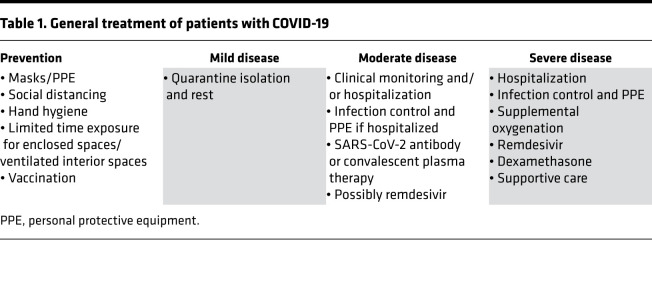
General treatment of patients with COVID-19

**Table 2:**
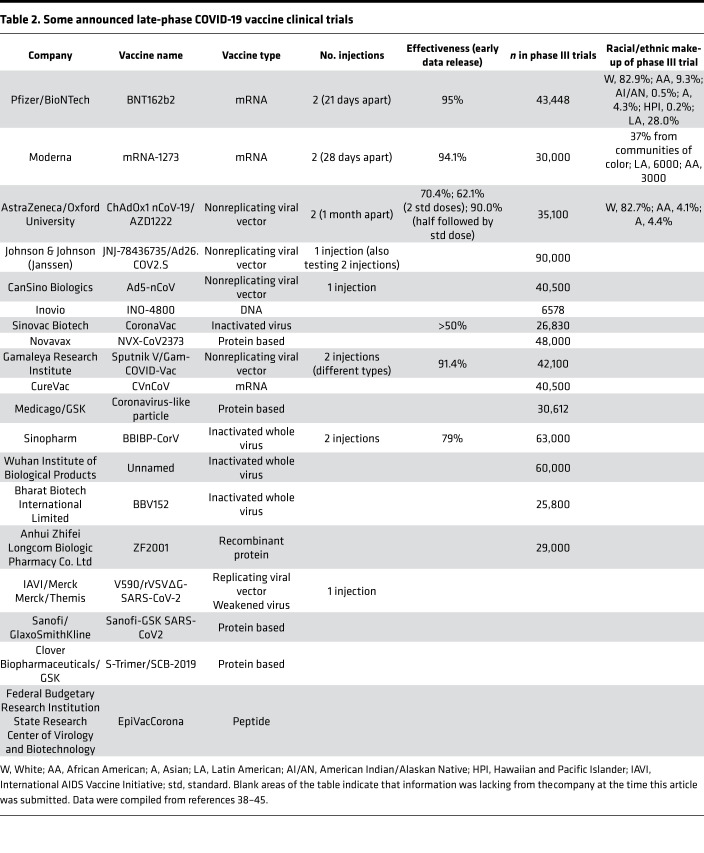
Some announced late-phase COVID-19 vaccine clinical trials

**Table 3:**
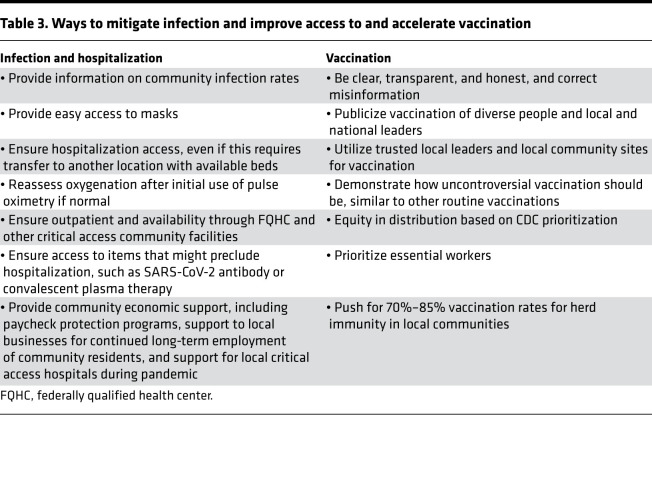
Ways to mitigate infection and improve access to and accelerate vaccination

## Version 1 01/21/2021

In-Press Preview

## Version 2 02/22/2021

Electronic publication
